# A new intracellular bacterium, *Candidatus* Similichlamydia labri sp. nov. (Chlamydiaceae) producing epitheliocysts in ballan wrasse, *Labrus bergylta* (Pisces, Labridae)

**DOI:** 10.1007/s00203-014-1061-4

**Published:** 2014-11-22

**Authors:** Andreas Steigen, Egil Karlsbakk, Heidrun Plarre, Kuninori Watanabe, Aina-Cathrine Øvergård, Øyvind Brevik, Are Nylund

**Affiliations:** 1 Department of Biology, University of Bergen, Bergen, Norway; 2 Institute of Marine Research, Bergen, Norway; 3 Sea Lice Research Center (SLRC), Institute of Marine Research, 5817 Bergen, Norway

**Keywords:** Epitheliocystis, Chlamydiales, Actinochlamydiaceae, Similichlamydia labri, Labridae, *Labrus bergylta*

## Abstract

Certain wrasse species (Labridae) are used as cleaner fish in salmon farms on the Norwegian coast, reducing salmon louse intensities. The pathogen repertoire of wrasse in Norway is poorly known, and the objective of the present study is to describe a novel intracellular bacterium detected in Norwegian *Labrus bergylta*. Histological examination of gill tissues from ballan wrasse, *L. bergylta,* revealed epitheliocysts occurring basally to the secondary lamellae in the interlamellar epithelium. Ultrastructurally, these had bacteria-filled inclusions with thickened membranes and radiating ray-like structures (actinae). 16S rRNA gene sequences from the gill bacteria showed the highest (97.1 %) similarity to *Candidatus* Similichlamydia latridicola from the gills of the latrid marine fish *Latris lineata* in Australia and 94.9 % similarity to *Candidatus* Actinochlamydia clariae, causing epitheliocystis in the freshwater catfish *Clarias gariepinus* in Uganda. A total of 47 gill samples from *L. bergylta* from Western Norway were screened by real time RT-PCR with an assay targeting *Candidatus* Actinochlamydiaceae 16S rRNA. Prevalence was 100 %. We propose the name *Candidatus* Similichlamydia labri sp. nov. for this new agent producing gill epitheliocysts in *L. bergylta*.

## Introduction

The ectoparasitic salmon louse, *Lepeophtheirus salmonis*, is a serious problem to farming of Atlantic salmon, *Salmo salar*, in Norway (Heuch et al. 2005). Among other control strategies, salmon farmers in Western Norway use wrasse (Labridae*)* as cleaner fish to reduce lice levels and the harmful consequences due to *L. salmonis*. In Western Norway, salmon famers mainly stock four, wild-caught, wrasse species with their salmon: *Labrus bergylta* (ballan wrasse), *Ctenolabrus rupestris* (goldsinny), *Symphodus melops* (corkwing wrasse) and *Centrolabrus exoletus* (rock cook). The fish is transported over long distances on the Norwegian coast, raising concerns about the possible spread of pathogens to previously uninfected wrasse populations. To gain knowledge on naturally occurring pathogens in wrasse, a study of gill-associated bacteria and parasites was initiated in 2011. It was found that epitheliocysts were common in the gills of *L. bergylta* in Western Norway.

Epitheliocysts have been observed on the gills in many fish species. In most cases, these show a chlamydia-like intracellular development (cf. Nylund et al. 1998). The rRNA gene sequences also usually suggest membership within Chlamydiales (Draghi et al. 2004, 2007; Meijer et al. 2006; Nowak and LaPatra 2006; Karlsen et al. 2008; Horn 2008; Polkinghorne et al. 2010; Schmidt-Posthaus et al. 2011; Corsaro and Work 2012; Fehr et al. 2013; Steigen et al. 2013; Stride et al. 2013a, b). Exceptions include a betaproteobacterium, found to cause epitheliocystis in farmed Atlantic salmon (Toenshoff et al. 2012; Mitchell et al. 2013), and a gammaproteobacterium responsible for epitheliocystis in cobia *Rachycentrum canadum* larvae (Mendoza et al. 2013).

In this study, we describe and characterise a new species of chlamydia detected in the gills of ballan wrasse (*Labrus bergylta*) from Western Norway. This new species show unique ultrastructural characteristics, resembling those seen in epitheliocysts in the African sharptooth catfish, *Clarias gariepinus*, from freshwater in Uganda (Steigen et al. 2013).

## Materials and methods

### Fish

Specimens of ballan wrasse, *Labrus bergylta,* were collected during May–June 2012 and May 2013 in Raunefjorden close to Bergen, Western Norway. These fish were mature or had just spawned. An autumn sampling period was in October 2012. Farmed ballan wrasse (*N* = 16) were also obtained from two different brood stock populations in Western Norway. The studied ballan wrasse caught in May 2013 measured 20–26 cm (*N* = 15), in June 2012, 21–41 cm (*N* = 27), and in October 2012, 27–37 cm (*N* = 4). The fish were caught with fyke nets and transported live to the laboratory at the University of Bergen. Gill samples were taken from newly killed fish, and frozen at −20 °C (for DNA/RNA) or fixed (4 °C) for histology including transmission electron microscopy (TEM).

### Histology and transmission electron microscopy

Gill tissue samples were immersed and fixed in a modified Karnovsky’s fixative: distilled water replaced by Ringer’s solution where sucrose was added (4 % w/v solution) (Nylund et al. 1998). The fixed tissues were used for histological studies and TEM, and were processed as described in Steigen et al. (2013).

### PCR/RT-PCR

RNA was extracted from individual gill samples of all specimens from each species sampled (as in Steigen et al. 2013). The RNA was used for real time RT-PCR detection of chlamydiae belonging to the *Candidatus* family Actinochlamydiaceae using the ChV assay (Steigen et al. 2013). DNA was extracted from selected samples with low cycle threshold values (Ct), since these contain larger amounts of bacteria. The 16S rRNA gene, the ITS region, and the partial 23S of the *Candidatus* Actinochlamydiaceae present were sequenced from five ballan wrasse selected due to low Ct values obtained by the ChV assay. A second type of chlamydia (clade A) was detected in six ballan wrasse individuals by PCR. The ChV assay does not amplify the clade A chlamydia. The PCR was performed as described by Steigen et al. (2013). Primers used are presented in Table 1.

**Table 1:** Primers used for PCR and sequencing of the 16S rRNA gene and ITS from *Candidatus* Actinochlamydiaceae obtained from the gills of wrasse

Primer	Sequence	Target gene	Publication
Pic-F1	AAG CAC TTT TGC CTG GGA GC	16S	Present study
Chits-F1	GGA ATT GCT AGT AAT GGC G	ITS	Present study
Chits-R1	TGG TCT CCC CAG ATT CAG ACS	ITS	Present study
Chits-R2	GTC TCC CCA GAT TCA GAC CG	ITS	Present study
16sigF	CGG CGT GGA TGA GGC AT	16S	Draghi et al. (2004)
806R	GGA CTA CCA GGG TAT CTA AT	ITS	Draghi et al. (2004)
16sB1	TAC GGY TAC CTT GTT ACG ACT T	16S	Draghi et al. (2004)

### In situ hybridization

In situ hybridization was performed as described by Dalvin et al. (2013), with some modifications presented in Tröße et al. (2014). The digoxigenin labelled (DIG-labelled) sense and antisense RNA probes (776 nucleotides) were made with primers: 16SIGF and 806R (Draghi et al. 2004).

### Phylogeny

The sequence data were preliminarily identified by GenBank searches done with BLAST (2.0). The Vector NTI Suite software package was used to obtain multiple alignments of sequences. Pairwise comparison of 16S sequences was done in GeneDoc (available at: www.psc.edu/biomed/genedoc). Selected sequences from other members of order Chlamydiales, available on the EMBL nucleotide database, were included in the comparisons. Phylogenetic analyses were performed using TREE-PUZZLE 5.2 (available at: http://www.tree-puzzle.de), maximum likelihood (ML) (50,000 puzzling steps). The data were analysed using a GTR-I nucleotide evolution model, selected in jModelTest; with default settings and the Akaike information criterion option (Posada 2008). Phylogenetic trees were drawn using TreeView (Page 1996).

### Ethics statement

All fish were treated according to the Norwegian Animal Welfare Act (01. 01. 2010) following regulations from the Norwegian Food Safety Authority.

## Results

### Occurrence of chlamydiae in *Labrus bergylta*

Screening (16S rRNA sequences) of gill tissues were carried out on 47 *L. bergylta*. PCR showed a 100 % prevalence of *Candidatus* Actinochlamydiaceae infections with Ct values always below 30. In May, samples average Ct was 18.5 (range 15.2–21.5), in June 21.4 (14.4–29.3), and in October 16.8 (15.0–18.1). Sequencing of the 16S rRNA gene from five samples provided identical or nearly identical sequences (identity 99.9–100.0, 2,102 nt compared) (type-A; accession nos: KC469556-8, KC469562, KC469564), with the highest affinity to *Candidatus* Similichlamydia latridicola.

Ballan wrasse was also found infected with a second chlamydia species (clade A), detected through PCR with general primers for Chlamydiaceae. The clade A (accession nos: KC469554-5, KC469559-61, KC469563) sequences showed the highest affinity to *Candidatus* Parilichlamydia carangidicola. Fish found infected with the clade A type of chlamydia were not used in the histological studies; the sequences, however, were included in the phylogeny.

### Bacteria and inclusion morphology

**Fig. 1:**
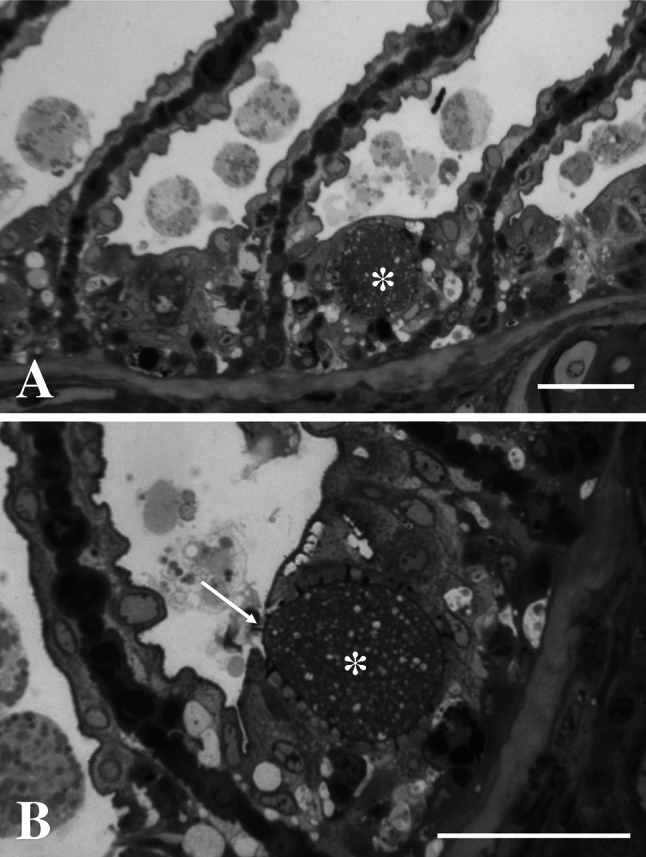
*Candidatus* Similichlamydia labri sp. nov. from the gills of *Labrus bergylta,* semi-thin sections stained with toluidine *blue*. **a** Epitheliocyst (*asterisk*) with typical site in an interlamellar crypt. Actinae can be seen radiating from the inclusion membrane. *Bar* = 25.0 µm. **b** An epitheliocyst with radiating actinae, opening (*arrow*) on the surface of the gills basal to the secondary lamellae. *Bar* = 30.0 µm (colour figure online)

**Fig. 2:**
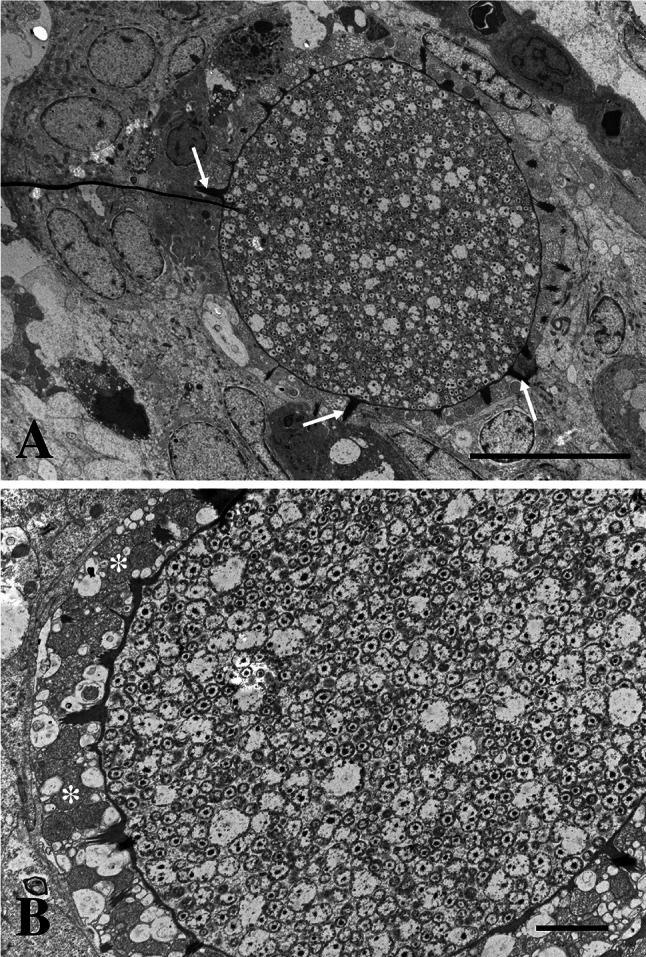
*Candidatus* Similichlamydia labri sp. nov. from the gills of *Labrus bergylta*, TEM. **a** An epitheliocyst between two secondary lamellae showing a round inclusion containing bacteria and an inclusion membrane with radiating actinae (*arrows*). There is no significant reaction in the surrounding tissue. *Bar* = 10 µm. **b** Inclusion containing bacteria with different morphologies (RB-like and IB-like morphs). The actinae penetrate the host cell cytoplasm, which contains large amounts of mitochondria (*asterisks*). *Bar* = 2.0 µm

**Fig. 3:**
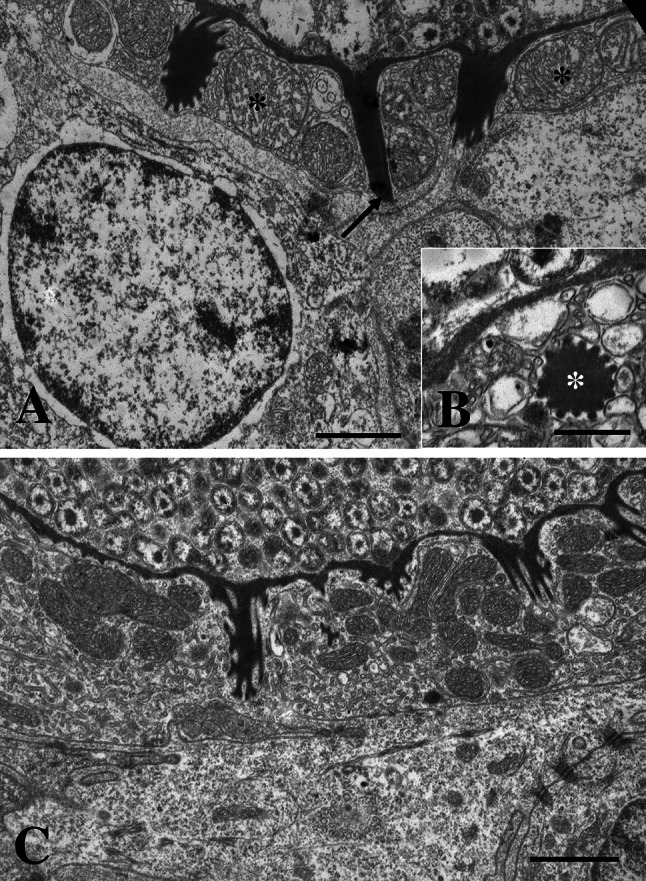
*Candidatus* Similichlamydia labri sp. nov. from the gills of *Labrus bergylta*, TEM. **a** High magnification of the actinae penetrating the host cell cytoplasm and into the neighbouring cell membrane (*arrow*). Mitochondria indicated by asterisks. *Bar* = 1.0 µm. **b** Transverse section of an actinia with sprocket-like morphology (*asterisk*). *Bar* = 0.5 µm. **c** A large amount of mitochondria and endoplasmic reticulum is present in the host cell cytoplasm between the actinae. *Bar* = 1.0 µm

Epitheliocysts in infected *L. bergylta* had a distinctive morphology (Fig. 1). The cysts occurred basal to the secondary lamellae, in cells resembling chloride cells (mitochondria and ER rich cells). The inclusions had numerous actinae radiating from the inclusion membrane (IM). Cysts in contact with the gill surface, reached 30 µm in diameter, but most inclusions measured <25 µm. Ultrastructurally, the IM was thickened and electron dense with regularly spaced actinae radiating from it (Fig. 2). The thickness of the IM was 50–100 nm, being thickest close to the actinae. The actinae consisted of the same electron dense material as the IM and showed longitudinal ridges on the surface producing a characteristic star-like pattern in transverse sections (Fig. 3). There was a central evagination of the inclusion vacuole into each actina, but the actinae did not appear to be hollow throughout. Near the IM, the actinae reached c. 650 nm in diameter, with up to 17 ridges, but some were smaller with only 6–7 ridges. The diameter and number of ridges tended to decrease with increasing distance from the inclusion. The actinae might reach to and apparently penetrate neighbouring cells; in some cases, the plasma membrane of the neighbouring cell disappeared in the contact region (Fig. 3a). The length of the actinae was typically 1.3–1.8 µm, but could reach 2.1 µm. The cytoplasm of the host cell was filled with mitochondria and membrane structures (endoplasmic reticulum), which occurred among the actinae and close to the IM.

The bacteria inside the inclusions were polymorphic and varied from irregular shapes with several nucleoids or with no distinct nucleoids [reticulate body (RB)-like morphology] to coccoid shapes with distinct electron dense nucleoids [intermediate body (IB)-like morphology]. The irregular and electron lucent RBs could exceed 2 µm in length. The coccoid cells had a diameter of about 200–360 nm with a nucleoid diameter of about 100 nm, some cells larger than 300 nm were seen dividing. The smallest coccoid cells had a more condensed cytoplasm, and they were not seen dividing and measured 200–250 nm in diameter [possibly an early stage of elementary bodies (EB)]. A cell wall and a cytoplasmic unit membrane surrounded all morphs of the bacteria inside the inclusions, but the cell wall was more distinct in the coccoid cells.

There was no sign of a host response (inflammation, hyperplasia) associated with the epitheliocysts in ballan wrasse.

### In situ hybridization

**Fig. 4:**
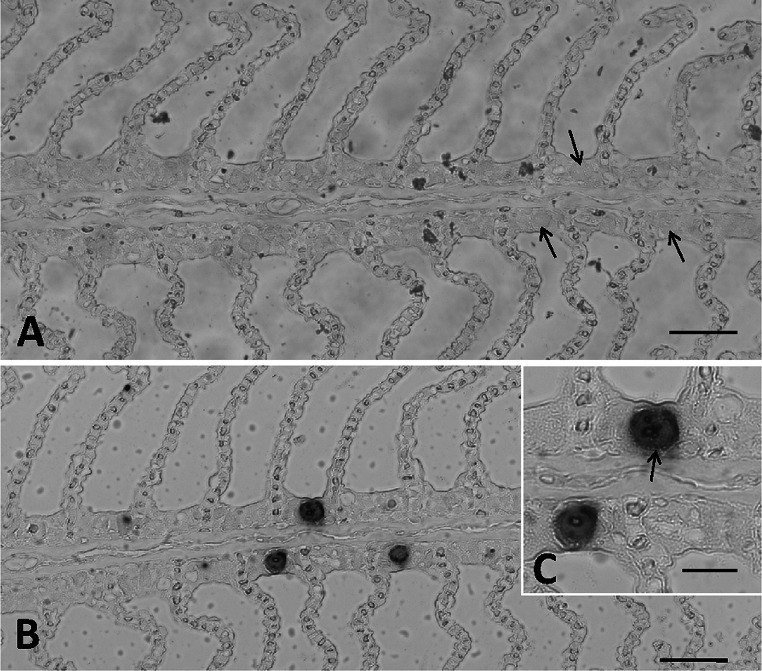
Sections of gill tissue from *Labrus bergylta* processed for in situ hybridization. **a** Primary gill filament stained with a sense probe, showing the absence of staining of the inclusions (*arrows*). *Bar* = 50.0 µm. **b** Primary filament showing *dark*–*blue* stained *Candidatus* Similachlamydia labri inclusions, stained with antisense DIG-labelled RNA probe against 16S rRNA of the bacterium. *Bar* = 50.0 µm. **c** Magnification of the IHC stained inclusion in figure B with discernible actiniae (*arrow*). *Bar* 20.0 = µm (colour figure online)

Inclusions in cells in the interlamellar epithelium of *Labrus bergylta* were specifically labelled with the 776 nt antisense riboprobe that was transcribed from 16S rRNA (accession no: KC469556) amplified from gills of the fish host. The ISH positive infected individual was among those studied by histology and TEM. Inclusions in adjacent histological sections that were incubated with the sense probe (776 nt) were not labelled (Fig. 4).

### Phylogeny and sequence comparisons

**Fig. 5:**
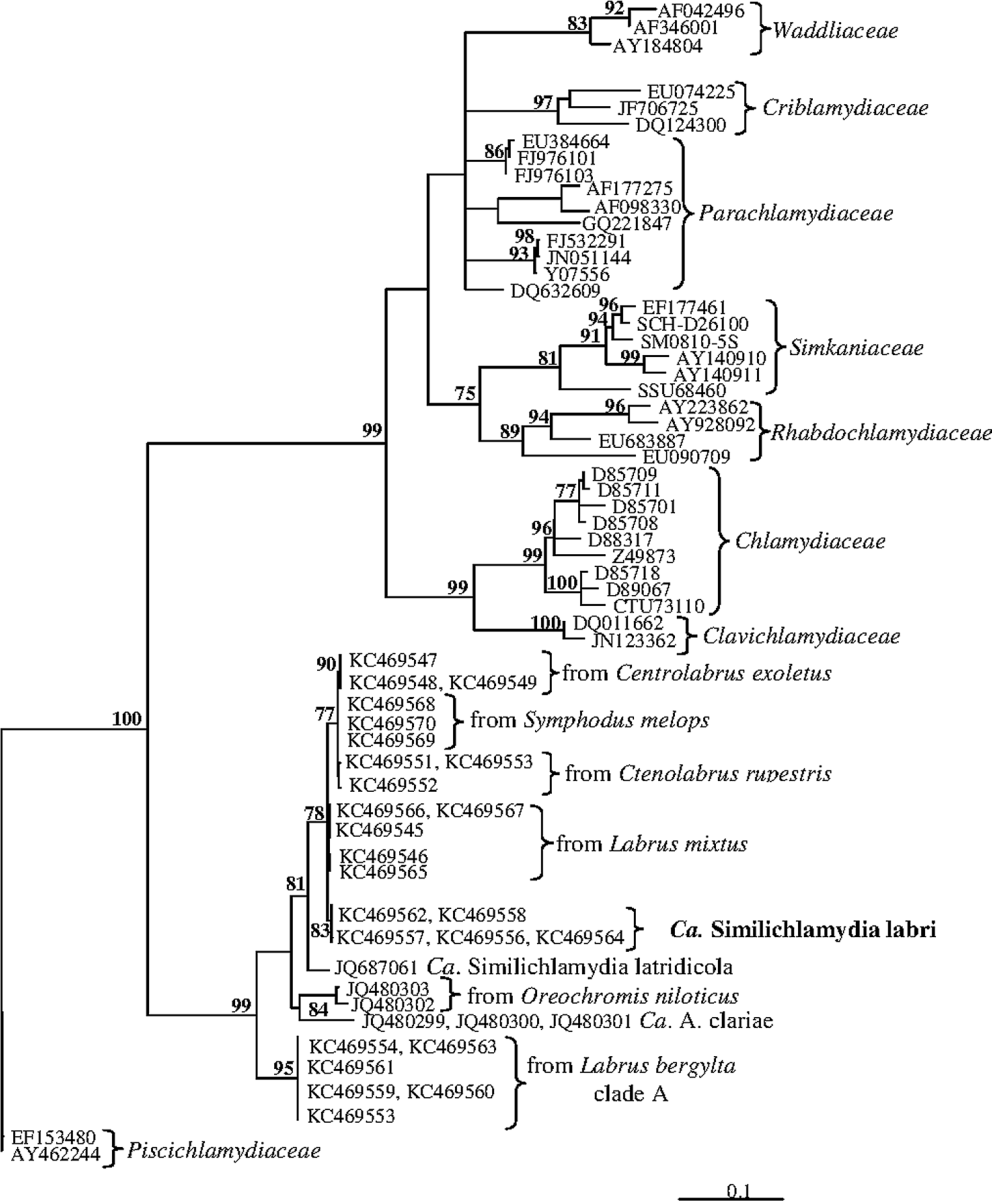
Phylogenetic tree showing the relationship between *Candidatus* Similichlamydia labri sp. nov. from *L. bergylta* and selected members of other families within order Chlamydiales. The analysis is based on a 1,210 nt long edited alignment of 16S rDNA sequences. The *scale bar* shows the number of nucleotide substitutions as a proportion of branch lengths. *Ca.* = *Candidatus*

**Fig. 6:**
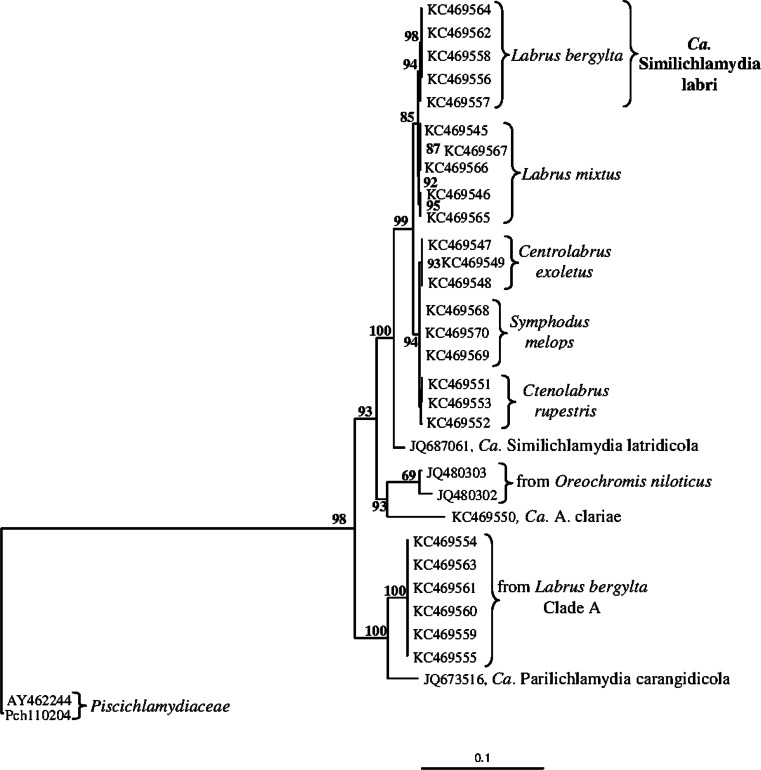
Phylogenetic tree showing the relationships between possible members of the family *Candidatus* Actinochlamydiaceae. Sequences of 16S rRNA gene sequences from *Candidatus* Piscichlamydia salmonis have been used as outgroup. The analysis is based on a 1,103 nucleotides long alignment of 16S rRNA gene sequences. The *scale bar* shows the number of nucleotide substitutions as a proportion of branch lengths. *Ca.* = *Candidatus*

Phylogenetic analyses showed that the 16S rRNA gene sequences of chlamydiae from ballan wrasse with epitheliocysts group with related chlamydiae from other Norwegian wrasse species into a major clade together with three previously described species: *Candidatus* Similichlamydia latridicola from striped trumpeter (*Latris lineata*) from a Tasmanian aquaculture facility, *Candidatus* *A. clariae* from *C. gariepinus* collected in fresh water in the vicinity of Lake Victoria, and *Candidatus* Parilichlamydia carangidicola from yellowtail kingfish (*Seriola lalandi*) in Australian waters (Figs. 5, 6). Two additional sequences obtained from the gills of *Oreochromis niloticus* (JQ480302, JQ480303) collected in Uganda also belong to this major clade (Fig. 5).

BLAST search and sequence comparisons revealed that sequence similarity of the chlamydiae to *Candidatus* S. latridicola is 97.1 % (1,395 nt compared), to *Candidatus* A. clariae 94.9 % (1,464 nt compared), and to *Candidatus* P. carangidicola 93.3 % (1,103 nt compared). The clade A sequences from ballan wrasse showed 93.8 % similarity with the chlamydia sequences (1,464 nt compared) from ballan wrasse with the described epitheliocysts. Phylogenetically, the two types grouped into two subclades (Fig. 5). One type grouped with *Candidatus* S. latridicola and *Candidatus* A, clariae, while the second type (clade A) grouped with *Candidatus* P. carangidicola (Fig. 6).

## Discussion

None of the Chlamydiales associated with epitheliocystis in fish have been cultured. New species are proposed as *Candidatus* species, based on the uniqueness of the 16S rRNA gene sequences, ultrastructure, and host specificity. A 16S rRNA gene sequence divergence of 1.0–1.3 % may separate different species (Stackebrandt and Ebers 2006; Kim et al. 2014).

The cysts and inclusions in infected *L. bergylta* showed similarities to those found in *Candidatus* Actinochlamydia clariae described from the gills of the African sharptooth catfish *Clarias gariepinus* in Uganda (Steigen et al. 2013). Ultrastructurally, they share a thickened electron dense inclusion membrane and the presence of actinae. The inclusions and the epitheliocysts reach a slightly larger size in ballan wrasse than in *Candidatus* A. clariae in catfish (30 vs. 23 µm in diameter). However, *Candidatus* Similichlamydia latridicola is more closely related to the *L. bergylta* chlamydiae than *Candidatus* A. clariae according to the 16S rRNA sequences. This species produce large cysts (>50 µm) in the secondary lamellae in the gills of *Latris lineata*. However, actinae have not been reported from *Candidatus* S. latridicola and are not evident from images in Stride et al. (2013b), and the ultrastructure of that candidate species is unknown.

Another difference between the epitheliocysts from infected ballan wrasse and *Candidatus* A. clariae is the appearance of the host cell cytoplasm. The inclusions of both chlamydiae were surrounded by a limited volume of host cell cytoplasm containing mitochondria and endoplasmic reticulum (cf. Steigen et al. 2013). However, a layer of vacuoles often surrounded the inclusions of *Candidatus* A. clariae setting them apart from the rest of the host cell (Steigen et al. 2013). Such vacuoles were not common around the inclusions from ballan wrasse.

We did not observe bacterial stages in the cysts from ballan wrasse similar to the elementary bodies (EB) of *Candidatus* A. clariae. The EBs from the latter contains a ‘polar cap area’ penetrated by rod-like structures arranged in a hexagonal pattern and protruding from the surface of the cap, and an eccentric nucleoid (Steigen et al. 2013). The lack of mature EB’s in the epitheliocysts from the studied ballan wrasse prevents a thorough comparison with the developmental stages of *Candidatus* A. clariae. However, further sampling should solve this issue.

The 16S rRNA gene sequences of the *L. bergylta* chlamydiae show 94.9 % similarity to those from *Candidatus* A. clariae. This suggests that they belong to the same family, but to a separate species in a different genus (cf. Everett 2000; Bush and Everett 2001; Stackebrandt and Ebers 2006). Based on 16S RNA gene sequence similarity (97.1 %), the *L. bergylta* chlamydia is congeneric with *Candidatus* S. latridicola. The clade A sequences obtained from *L. bergylta* from Western Norway (accession no: KC469554-5, KC469559-61, KC469563) and *Candidatus* P. carangidicola (accession no: JQ673516) from Australian waters are the nearest relatives to the *Ca.* Actinochlamydiaceae, grouping in a separate clade (Fig. 5). The morphology of the clade A chlamydiae is unknown.

The epitheliocyst producing agent found and described in the present study shows a close relationship to species found in both fresh- and seawater, in cold and in warm regions. The wide geographical distribution is remarkable, with related species off Australia and Norway, and in Lake Victoria. A high global diversity therefore seems likely for these parasitic bacteria, meriting further investigations. Material now being processed in our laboratory will expand this distribution further and add new *Candidatus* species.

We suggest that the described chlamydia from *Labrus bergylta* is given status as a new species, *Candidatus* Similichlamydia labri n.sp. The 16S RNA gene sequences, site, host, and geographic location clearly distinguish it from *Candidatus* Similichlamydia latridicola.

## Taxonomy

*Candidatus* Similichlamydia labri [labr’i M.L. gen. sing. of *Labrus*, generic name for the fish host *Labrus bergylta*; of (living in) members of the genus *Labrus*].

- Type host: *Labrus bergylta* Ascanius (Labridae)
- Site: intracellular inclusions in chloride cells in the gills
- Type locality: Raunefjorden, near Bergen, Hordaland County, Norway (60°16′N, 05°13′E)
- 16S and ITS rDNA Sequence: GenBank Acession No. KC469556

The provisional taxon *Candidatus* Similichlamydia labri represents an intracellular bacterium within membrane-bound vacuoles (inclusions) in the cytoplasm of chloride cells in the gills of *Labrus* *bergylta*. Inclusion membranes are thickened, ridged, and give rise to ridged actinae. The developmental stages include RBs of varying size, shape and number of nucleoids, and smaller coccoid cells, <300 nm in diameter. The bacterium cell wall is thin; typical EBs have not been observed.
